# Unilateral Eyelid Edema as Initial Sign of Orbital Sarcoidosis

**DOI:** 10.1155/2016/6912927

**Published:** 2016-05-19

**Authors:** Sílvia Miguéis Picado Petrarolha, Bruna Suda Rodrigues, Flávio David Haddad Filho, Rogério Aparecido Dedivitis, Samuel Brunini Petrarolha, Pedro Martins Tavares Scianni Morais

**Affiliations:** ^1^Department of Head and Neck Surgery, Hospital Ana Costa, 11050-120 Santos, SP, Brazil; ^2^Universidade Metropolitana de Santos (UNIMES), 11050-120 Santos, SP, Brazil; ^3^Department of Head and Neck Surgery, Hospital das Clínicas, University of São Paulo School of Medicine, São Paulo, SP, Brazil; ^4^Department of Head and Neck Surgery, Hospital Ana Costa, Santos and Irmandade da Santa Casa da Misericórdia, 11050-120 Santos, SP, Brazil; ^5^Department of Ophthalmology, Hospital Ana Costa, 11050-120 Santos, SP, Brazil

## Abstract

*Introduction*. Sarcoidosis is a rare multisystemic granulomatous inflammatory disease of unknown etiology affecting the respiratory system, skin, and eyes. Sarcoidosis outside the lacrimal gland is rare. The case study concerns a patient with a final diagnosis of orbital sarcoidosis.* Case Report*. A 37-year-old male patient went to the ophthalmic emergency room complaining of pain in the left eye, diplopia, and decreased visual acuity. An external eye examination showed hard and cold edema of the lower eyelid, ocular motility with limitation of adduction, and discreet ipsilateral proptosis. Magnetic resonance of the orbit showed left eye proptosis and thickening and increase of soft tissues associated with heterogeneous impregnation of contrast in the infralateral region of the left eyelid. A biopsy of the lesion showed a chronic inflammatory process, with numerous compact nonnecrotizing granulomas surrounded by lamellar hyaline collagen, providing histological confirmation of sarcoidosis.* Discussion*. A biopsy of the orbital tumor is essential for the diagnosis of sarcoidosis, in addition to the search for systemic findings such as hilar adenopathy or parenchymal lung disease found in 90% of patients.

## 1. Introduction

Sarcoidosis is a rare multisystemic, chronic, granulomatous inflammatory disease of unknown etiology that affects the respiratory system, skin, and eyes [1–4]. It is characterized by noncaseating granulomas with no lymphocytic border and it is more common in people of Afro-Caribbean descent, during the 6th decade of life, and in women, for unknown reasons [1, 4–7].

Bilateral hilar lymphadenopathy and skin or eyelid lesions are the most common symptoms in sarcoidosis. Ocular involvement occurs in about 25% to 50% of patients, with the lacrimal gland being the most common site. Sarcoidosis with orbital presentation outside the lacrimal gland is rare in all age groups [1, 7, 8].

The case study concerns a patient with a final diagnosis of orbital sarcoidosis.

## 2. Case Report

**Figure 1:**
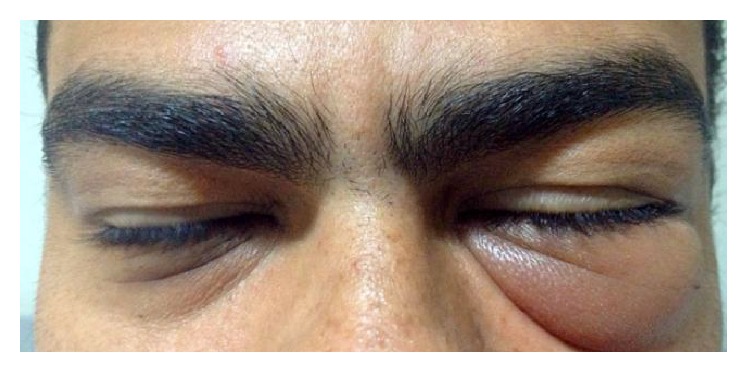
Hard and cold edema of the lower left eyelid with discreet ipsilateral proptosis.

A 37-year-old male patient went to the ophthalmic emergency room complaining of pain in the left eye, lasting for two months, with high intensity shooting and progressive ipsilateral eyelid swelling, followed by diplopia and decreased visual acuity. An external eye examination showed hard and cold edema of the lower eyelid, ocular motility with limitation in adduction, and discreet ipsilateral proptosis. Hertel exophthalmometry was used to measure the degree of ocular protrusion and the value obtained was 18,2 mm for the right eye and 25 mm for the left eye (Figure 1).

The best corrected visual acuity was full (20/20) in the right eye and 0.8 (20/25) in the left eye. No refractive error was detected in any of the eyes. Biomicroscopic examination of the right anterior segment did not reveal any abnormal findings, although the left eye showed conjunctival hyperemia, chemosis, and keratitis.

**Figure 2:**
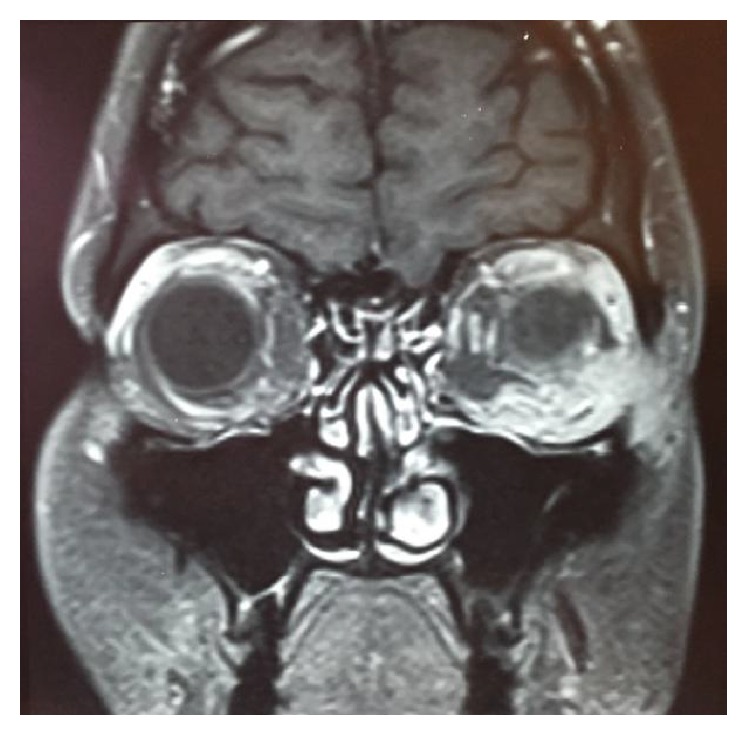
Coronal T1 fat-saturated postgadolinium: solid mass enhancing the gadolinium, located within retroconal, pre- and post-septal, and inferolateral regions.

**Figure 3:**
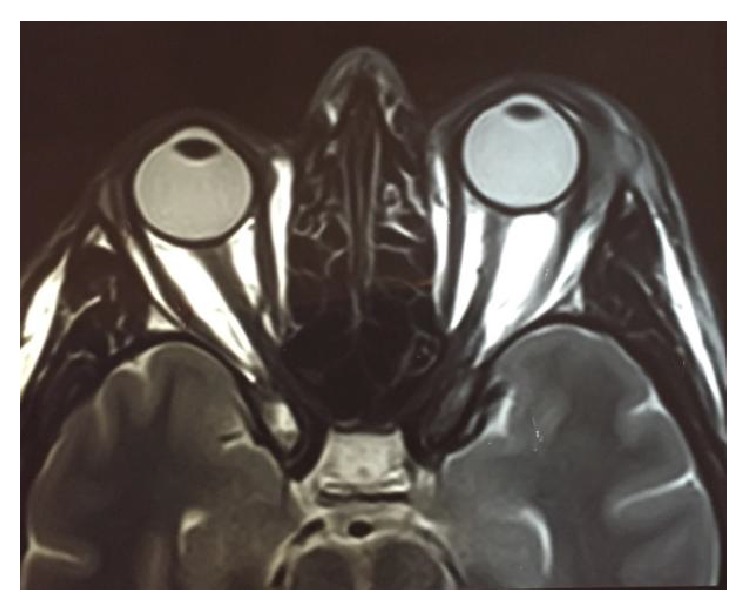
Axial T2-anterior projection of the left eye globe in relation to the contralateral.

Intraocular pressure was measured using an applanation tonometer and it was found to be 11 mmHg in the right eye and 16 mmHg in the left eye. No pathological findings were detected during fundoscopy. The optical coherence tomography (OCT) of the left eye revealed no abnormalities. The cause for low vision in the left eye was found to be eyelid edema and keratitis. The cause for left eye motility reduction was the extraocular muscles (EOM) affected by the lesion. The affected EOM were the lateral rectus, inferior rectus, and inferior oblique. Magnetic resonance of the orbit showed left eye proptosis and thickening and increase of soft tissues associated with heterogeneous impregnation of contrast in the infralateral region of the left eyelid (Figures 2 and 3). A biopsy was performed in the lower left eyelid on the second day of hospitalization using the Posterior Inferior Orbitotomy to access the lesion.

**Figure 4:**
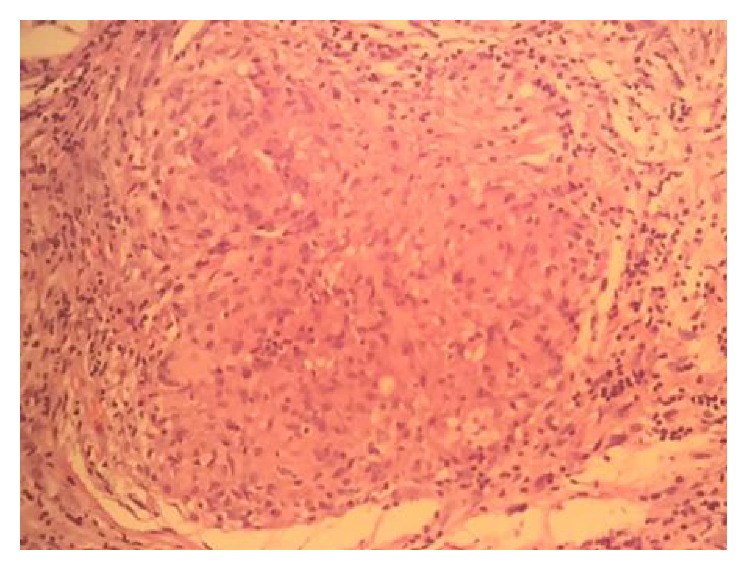
Extensive multifocal chronic granulomatous inflammation, with numerous epithelioid macrophages and multinucleated giant cells, without evidence of necrosis (HE 100x).

Histology showed a chronic inflammatory process, well-formed, tightly packed, with nonnecrotizing granulomas surrounded by lamellar hyaline collagen, and negative immune-histochemistry for mycobacteria, providing histological confirmation of sarcoidosis (Figure 4). Computed tomography showed enlargement of the pulmonary hilar as a result of bilateral lymphadenopathy. AST was 450 U/L (reference value: 5–40 U/L) and ALT was 375 U/L (value: 7–35 U/L), both higher than usual. Serum angiotensin converting enzyme (ACE) levels were also above the normal levels, 145 mg/dL (reference value: <56 mg/dL). The patient was referred to the Rheumatology Service, which carried out endovenous pulse therapy with methylprednisolone 1 g a day for five days. Keratitis was treated with lubricant sodium hyaluronate based eye drops. 1 drop was applied in the left eye every 4 hours during the time the patient remained in hospital. After treatment with pulse therapy, the patient showed remission of the eyelid edema and chemosis.

During ophthalmologic treatment at the time of discharge, right eye acuity was 20/20 and visual acuity at the left eye went back to normal standards. The left eye movement was totally restored. Fundoscopy showed no change in either eye. Eye pressure was 13 mmHg for the right and left eye. The patient was discharged after significant improvement of his ophthalmologic condition and with no pain. The patient was reassessed 7, 15, 30, 45, and 60 days after hospital discharge, having presented no pathological changes in the ophthalmologic exam, with complete remission of the disease.

## 3. Discussion

Orbital inflammatory lesions cover a number of hypotheses, such as vasculitis, sarcoidosis, orbital myositis, Graves' ophthalmopathy, autoimmune thyroid disease, foreign body, idiopathic orbital inflammation, other differential diagnoses, infections, neoplasia, and congenital lesions [3, 5, 9–11]. Sarcoidosis can affect almost any structure within or around the eye and its gold standard for diagnosis is a tissue biopsy [11, 12].

A biopsy of the orbital tumor is essential for the diagnosis of sarcoidosis and it must demonstrate histologic evidence of noncaseating granulomas. In addition, other systemic findings such as hilar adenopathy or parenchymal lung disease are present in 90% of patients [2, 3, 7, 9]. Furthermore, an increase in the ACE also assists in diagnosis, in combination with chest imaging [3, 7, 10, 12].

Sarcoidosis should always be considered in the differential diagnosis of orbital mass in all ages, even when there is no involvement of the lacrimal gland [1]. The treatment represented by corticosteroids (systemic therapy) is the same as for other inflammatory orbital pseudotumors, and it is extremely important as it can influence the functional ocular prognosis of patients [2]. Immunosuppressive agents such as methotrexate, mycophenolate mofetil, and azathioprine can also be used for long term control of systemic and ocular inflammation [7]. Surgical treatment is not recommended due to the likelihood of local recurrences and complications [11].
